# Testing the *What Matters to Me* workbook in a diverse sample of seriously ill patients and caregivers

**DOI:** 10.1016/j.pecinn.2023.100216

**Published:** 2023-09-16

**Authors:** Erik K. Fromme, Lauren Nisotel, Kimberly Mendoza, Ayush Thacker, Kurt Lowery, Bukiwe Sihlongonyane, Katherine O. DeBartolo, Jane Roessner, Judy N. Margo

**Affiliations:** a Serious Illness Care Program, Ariadne Labs, Boston, MA, USA; b Department of Psychosocial Oncology and Palliative Care, Dana-Farber Cancer Institute, Boston, MA, USA; c American Cancer Society, Atlanta, GA, USA; d Harvard T.H Chan School of Public Health, Boston, MA, USA; e Brandeis University, Waltham, MA; f Boston University School of Public Health, Boston, MA, USA; g The Conversation Project, Institute for Healthcare Improvement, Boston, MA, USA; h Science & Technology Platform, Ariadne Labs, Boston, MA, USA

**Keywords:** Serious illness, Communication, Advance care planning, Patient education, Caregivers, Goal concordant care

## Abstract

**Objectives:**

We evaluated the *What Matters to Me* Workbook, a patient-facing version of the Serious Illness Conversation Guide co-created by Ariadne Labs and The Conversation Project.

**Methods:**

We purposively recruited diverse seriously ill patients and caregivers in the US. Participants completed the Workbook, a survey, and a semi-structured in-depth interview about their experience. Qualitative analysis of interviews and notes was employed to extract themes. Simple descriptive statistics were employed to analyze eight investigator authored questions.

**Results:**

Twenty-nine study participants completed twenty-one interviews and twenty-five surveys. Ratings for safety (3.87/4, SD = 0.43) and acceptability (3.59/4, SD = 0.956) were higher than ratings for ease of use (3.30/4, SD = 0.97) and usefulness (3.24/4, SD = 0.80). Qualitative analysis identified that while the workbook was safe, acceptable, easy to use, and useful, it is more important who is recommending it and how they are explaining it.

**Conclusion:**

If presented in the right way by a trustworthy person, the *What Matters to Me* Workbook can be an easy to use, useful, and safe resource for patients with serious illness and their caregivers.

**Innovation:**

The Workbook focuses on serious illness rather than end-of-life and meshes with a clinician-facing conversation guide and a health-system level intervention.

## 1 Introduction

Conversations about patients' values and goals in serious illness (“serious illness conversations”) often occur very late in the illness course or not at all, contributing to unnecessary suffering for patients and their caregivers and moral distress for clinicians—particularly when care is out of alignment with patients' priorities. Ariadne Labs is a center for health care delivery innovation between the Brigham and Women's Hospital and Harvard T.H. Chan School of Public Health in Boston, Massachusetts that is dedicated to finding solutions for “know-do” gaps, or the gaps between evidence-based best practice and actual practice. Ariadne Labs developed the *Serious Illness Conversation Guide* (the Guide) to help clinicians have productive, efficient, and patient-centered serious illness conversations and to enable the Serious Illness Care Program (the Program) to help health systems implement a population health approach to ensuring conversations happen consistently and early enough to make a difference [[1], [2], [3]]. While the Guide and the Program focus on preparing clinicians and health systems, less attention has been paid to preparing patients and their caregivers to engage in conversations using the Guide.

The Guide poses questions that patients may have not considered before, that may be intensely personal, and that may elicit strong emotions—e.g., “What are your most important goals if your health condition worsens?” and “If you become sicker, how much are you willing to go through for the possibility of gaining more time?” Some patients and their friends and family members (their important people) may have already been thinking and talking about these questions as part of advance care planning (ACP) or because the questions arise naturally when dealing with a serious, life-threatening illness [4]. Others may be avoiding thinking or talking about these questions because they trigger anxiety or grief or because the individuals involved are worried about upsetting each other [[5], [6], [7]].

Consequently, Ariadne Labs partnered with The Conversation Project, an initiative of the Institute for Healthcare Improvement, that specializes in designing and disseminating resources to help people have conversations about what matters most in their health care. Together, we created a patient-facing version of the Serious Illness Conversation Guide called the *What Matters to Me* Workbook to help seriously ill patients and their caregivers to contemplate and perhaps discuss where they stand before communicating their values and goals to their health care team. Many patient-facing resources exist to help people with end-of-life planning. The Workbook is innovative in two ways – first, it focuses on decision-making in serious illness rather than end-of-life planning. Second, rather than existing in a vacuum, the Workbook is paired with the well-established clinician-facing Serious Illness Conversation Guide.

The first version of the *What Matters to Me* Workbook (the Workbook) [8] was created using the nine questions from Ariadne Labs' Serious Illness Conversation Guide, supplemented with additional questions and content, much of it derived from The Conversation Project's *Conversation Starter Guide* [9]. The first step was to do an environmental scan of patient-facing ACP resources to create an options document. We reviewed design decisions from the options document with experts in ACP and serious illness communication, collecting their opinions during two online focus group discussions or in writing via a questionnaire. Their expert opinions and suggestions were incorporated into the first draft of the Workbook. The end result was an eight-page booklet, intended to be usable by a lay audience with or without support from a healthcare professional. This project aimed to test the Workbook with a diverse sample of patients with serious illness and their caregivers to evaluate and improve its acceptability, ease-of-use, safety, and usefulness.

## 2 Methods

### 2.1 Participant eligibility and recruitment

We initially sought to recruit patient/caregiver dyads where the patient affirmed they have a serious illness such as metastatic cancer, chronic kidney disease on hemodialysis, chronic lung disease, neurological disease, etc. Caregivers were defined as friends or family members of the seriously ill person who were routinely involved in conversations with their health professionals. Participants' eligibility included being English-speaking, 18 years or older, able to participate in an interview by telephone or video call, and able to provide verbal or written consent.

Participants were recruited between November 2021 and June 2022 during the COVID-19 pandemic. Participants who did not have a family caregiver, or did not want them involved could participate, however caregivers could not participate unless their “caregivee”/patient also agreed to participate as the patient would be the one completing the workbook. We began by asking our community partners who led local, regional, or national faith communities, patient support groups, or patient advocacy organizations across the United States to identify potential participants. We asked them ask potential participants' permission for a member of the research team (KM, AT, EKF) to contact them. Halfway through the study, we expanded our enrollment criteria to include “caregiving experts” who were interviewed individually rather than paired with a patient. These participants were colleagues and community partners, known to us from prior collaborations, who were actively working in serious illness care in underrepresented and minority (URM) communities as palliative care clinicians, implementers, researchers, or public policy advocates. This change was made after largely unsuccessful efforts to recruit URM participants. While not a perfect solution, this allowed us to broaden the diversity of communities from which we could learn.

### 2.2 Enrollment

Eligible individuals were contacted by phone by a team member (KM, EKF) to review the study and participation requirements, answer questions, obtain and document verbal consent, and schedule the interview. On average, two attempts were made to contact eligible prospective participants by phone and email.

### 2.3 Study procedure

As we wanted to assess their experience using the Workbook, patients and caregivers were asked to complete the Workbook and discuss their answers with each other prior to interview participation. Four caregiving experts were asked to review the Workbook. Two chose to give their own perspectives on the WB based on their clinical and research experience, whereas two also asked their family members for input and combined their own and family members' observations.

### 2.4 Ethical review

The study was reviewed and approved by Mass General Brigham Institutional Review Board.

### 2.5 Semi-structured interviews

Interviews lasted 45 to 60 min and were based on a guide soliciting the participants' experience using the Workbook, as well as, questions covering domains of acceptability, ease of use, safety, and usefulness of the Workbook. All interviews were conducted by one member of our team (EF) with notetaking by another member (AT, JM). Team members involved with interviewing and note-taking were either established qualitative researchers (EF, JM) or were trained by a senior team member (AT). All interviews were conducted via telephone or video call. Upon affirming verbal consent from participants, interviews were recorded. Interviews were then professionally transcribed, and speakers were labeled in transcripts as being either patients or caregivers.

In addition to recorded responses, the investigators recorded observations about the recruitment process, participant's challenges completing the Workbook, and investigator observations of completed workbooks when participants were able to share them. These observations are a fundamental element of usability testing, which should not rely solely on participants' self-report [10].

### 2.6 Surveys

Participants who completed interviews were asked to complete a web-based survey using Research Electronic Data Capture (REDCap) [11]. The survey included demographic questions and investigator-authored survey questions about the Workbook. We also included the nine-item version of Sudore's Advance Care Planning Engagement Survey, which assesses self-efficacy and readiness to name a surrogate decision maker and to discuss what kind of care they would want if they were seriously ill or at the end of life [[12], [13], [14], [15]]. Finally, we assessed the Net Promoter Score, a popularized measure of customer satisfaction and brand loyalty that asks users to rate how likely they are to recommend a product (in this case the Workbook) to a friend or relative on a scale from 0 (not at all likely) to 10 (extremely likely). Responses are categorized into three groups: “Detractors” (ratings 0–6), “Passives” (ratings 7 and 8) and “Promoters” (ratings 9 and 10). The overall “Net Promoter Score”, is calculated by subtracting the percentage of Detractors from the percentage of Promoters, therefore NPS can range from −100 (i.e., all Detractors) to +100 (i.e., all Promoters) [16].

### 2.7 Qualitative analysis

Thematic qualitative analysis was conducted using NVivo (released in March 2020). We developed a codebook reflecting our a priori domains: logistical aspects of Workbook use (e.g., time to complete); ease of use; acceptability of the Workbook (e.g., understandable language, relatability of examples); safety related to using the Workbook (e.g., did its use contribute to disruption or argument); emotions related to using the Workbook (e.g., feelings evoked or reactions to the fact that feelings are evoked); usefulness of engaging with the Workbook (e.g., benefits or value); actions completed or anticipated following Workbook completion; prior preparation (e.g., previous contemplation of ACP); and suggested changes to the Workbook. In addition to these deductive themes, the codebook was subsequently updated to include themes that emerged inductively from the data. The patient versus caregiver roles were considered as an aspect of analysis.

Coding was completed by two team members (JM, AT), who each coded the same two transcripts (10% of the total), meeting after each to discuss and resolve any dissimilarities in code application and understanding. Once initial intercoder agreement was established, they divided and coded the remaining transcripts individually, continuing to meet regularly to discuss and resolve any uncertainties. The resulting coded data were analyzed by three team members (EF, JM, AT), reviewing all excerpts by code and developing findings by theme and sub-theme.

### 2.8 Quantitative analysis

Quantitative analysis was done by one team member (LN) and consisted of percentages, means, and standard deviations calculated using IBM SPSS Statistics for Windows, version 27.0.

## 3 Results

### 3.1 Participants

Twenty-one interviews were conducted with 29 participants. Nine interviews were conducted with patient/caregiver pairs, four interviews with individual patients, three interviews with individual caregivers, and four interviews with caregiving experts. The average interview length was 55 min (ranging from 33 to 77 min). The transcripts ranged from 16 to 25 pages in length. Table 1 presents demographic information on 25 of the 29 participants. The four remaining participants were unable to complete the electronic surveys due to cognitive impairment (2), visual impairment (1), or lack of internet access (1).

**Table 1:** Personal demographic characteristics of study sample.

Variable	N (%)
Total	25⁎
Participant Type	
Patient	9 (36)
Caregiver	12 (48)
Both	1 (4)
Other	3 (12)
Relationship to Participant	
Partner/spouse	8 (32)
Parent	3 (12)
Child	8 (32)
Friend/roommate	2 (8)
Health Care Proxy/Agent/Decision Maker	2 (8)
Other	2 (8)
Age	
31–40 years	4 (16)
41–50 years	5 (20)
51–60 years	5 (20)
61–70 years	4 (16)
71–80 years	7 (28)
Sex	
Female	19 (76)
Male	6 (24)
Race	
Asian	1 (4)
Black or African American	4 (16)
White	18 (72)
Other	1 (4)
Prefer Not to Answer	1 (4)
Ethnicity	
Hispanic/Latinx	2 (8)
Not Hispanic/Latinx	23 (92)
Native English Speaker	
Yes	22 (88)
No	3 (12)
Education	
High school graduate or equivalent	1 (4)
Some college	1 (4)
College graduate	11 (44)
Graduate or professional school	12 (48)
Health Insurance	
Medicare	5 (20)
Medicare plus supplement	7 (28)
Medicaid	1 (4)
Private insurance	14 (56)
Uninsured	1 (4)
Other	3 (12)
Health Care Proxy	
Yes	18 (75)
No	6 (25)
Region	
Midwest	4 (16)
Northeast	12 (48)
South	5 (20)
West	4 (16)
Community	
Suburban	18 (72)
Urban	7 (28)
Living Situation	
Self/alone	7 (28)
Partner/spouse	17 (68)
Dependent children	4 (16)
Independent children	3 (12)
Dependent adult	1 (4)
Other	3 (12)
Religion	
Agnostic	2 (8)
Atheist	1 (4)
Born-again or Evangelical Christian	7 (28)
Catholic	5 (20)
Protestant	3 (12)
Unitarian/Universalist	1 (4)
Other	4 (16)
No religion	4 (16)
Attendance of Religious Services	
At least once a week	7 (28)
Once or twice a week	5 (20)
A few times a year	4 (16)
Seldom	2 (8)
Never	7 (28)

⁎ Four participants, all patients, completed a qualitative interview, but did not complete the cross-sectional survey and are not included in this table.

### 3.2 Quantitative survey findings

As expected, participants reported high levels of readiness and confidence in ACP (*M* = 4.28, *SD* = 0.85, range 1–5) (Table 2). Interestingly they reported being least ready to talk to their doctors – about appointing a medical decision maker (*M* = 3.87, *SD* = 1.49, range 1–5) or about the kind of medical care they would want if they were very sick or near the end of life (*M* = 3.57, *SD* = 1.59, range 1–5).

**Table 2:** Advance care planning engagement scale^a^, ^b^.

Item:	Mean (SD)
1. How ready are you to formally ask someone to be your medical decision maker?	4.46 (0.98)
2. How ready are you to talk with your DOCTOR about who you want your medical decision maker to be?	3.87 (1.49)
3. How ready are you to SIGN OFFICIAL PAPERS naming a person or group of people to make medical decisions for you?	4.33 (1.09)
4. How ready are you to talk with your DECISION MAKER about the kind of medical care you want if you were very sick or near the end of life?	4.13 (1.18)
5. How ready are you to talk with your DOCTOR about the kind of medical care you want if you were very sick or near the end of life?	3.57 (1.59)
6. How ready are you to SIGN OFFICIAL PAPERS putting your wishes about the kind of medical care you want if you were very sick or near the end of life?	4.43 (0.99)
7. How confident are you that today you could ask someone to be your medical decision maker?	4.67 (0.57)
8. How confident are you that today you could talk with your decision maker about the care you would want if you were very sick or near the end of life?	4.71 (0.69)
9. How confident are you that today you could talk with your doctors about the care you would want if you were sick or near the end of life?	4.39 (0.94)
Advance Care Planning Engagement Scale Overall Score	*4.28 (0.85)*

a Response range for “readiness” items #1-#6 includes 1 “I have I have never thought about it”; 2 “I have thought about it, but I am not ready to do it”; 3 “I am thinking about doing it in the next 6 months”; 4 “I am definitely planning to do it in the next 30 days”; or 5 “I have already done it”.

b Response range for “self-efficacy” items #7-#9 varies from 1 “Not at all confidant” to 5 “Extremely Confidant”.

Participants' ratings on the eight structured evaluation items are reported in Table 3. Overall, their ratings for safety (*M* = 3.87, *SD* = 0.43, range 0–4) and acceptability (*M* = 4.59, *SD* = 0.96, range 1–5) were higher than their ratings for ease of use (M = 4.53, SD = 0.97, range 1–5) and usefulness (*M* = 3.24, *SD* = 0.80, range 0–4). The Net Promoter Score [16] was calculated by responses to the question, “On a scale of 0 to 10, how likely are you to recommend the *What Matters to Me* Workbook to a friend or relative?” Our respondents included 13 “promoters” (rated 9 or 10, 56.5%), nine “passives” (rated 7 or 8, 39%), and one “detractor” (rated 0–6, 4.5%), for a Net Promoter Score of +52/100, which is generally considered excellent [17].

**Table 3:** Ease of use, acceptability, usefulness, and safety of the *What Matters to Me* workbook^a^.

Variable	N (%) (of 23 total)	Mean (SD)
Ease of Use		
The design of the Workbook made it easy to use.		3.30 (0.97)
Extremely	13 (57)	
Fairly	6 (26)	
Somewhat	2 (8.5)	
A little	2 (8.5)	
The language in the Workbook was easy to understand.		3.61 (0.58)
Extremely	15 (65)	
Fairly	7 (30)	
Somewhat	1 (5)	
*Total Ease of Use Score:*		*3.30 (0.97)*
Acceptability		
The Workbook asks questions that are important to think about now.		3.74 (0.45)
Extremely	17 (74)	
Fairly	6 (26)	
The Workbook is culturally appropriate to me.		3.59 (0.96)
Extremely	17 (74)	
Fairly	3 (13)	
Somewhat	1 (4.3)	
Not at all	1 (4.3)	
Not sure	1 (4.3)	
*Total Acceptability Score:*		*3.59 (0.69)*
Usefulness		
The Workbook helped me get more clear about what is important to me if I get sicker.		3.30 (0.88)
Extremely	13 (56.5)	
Fairly	4 (17.4)	
Somewhat	6 (26.1)	
The Workbook helped prepare me to talk to my family member/friend and my health care team (doctor, nurse, social worker, etc.) about what is most important to me.		3.17 (0.89)
Extremely	10 (43.5)	
Fairly	8 (34.8)	
Somewhat	4 (17.4)	
A little	1 (4.3)	
*Total Usefulness Score:*		*3.24 (0.80)*
Safety		
Completing the Workbook was more upsetting than worthwhile.		3.83 (0.49)
Fairly	1 (4.3)	
A little	2 (8.7)	
Not at all	20 (87)	
I regret trying the Workbook.		3.91 (0.42)
Not at all	22 (95.7)	
Fairly	1 (4.3)	
*Total Safety Score:*		*3.87 (0.43)*

^b^ Overall scores for each domain were calculated by averaging the two individual item scores for each domain.

a Response range for items from 0 “not at all”; 1 “a little”; 2 “somewhat”; 3 “fairly”; 4 “extremely”.

### 3.3 Qualitative interview findings

#### 3.3.1 Ease of use

In general, all participants, including URM, were positive about the Workbook. The comparatively lower ratings for ease of use and usefulness noted above were explained in the interviews. Regarding ease of use, by far the most common complaints were related to technical difficulties completing the PDF-formatted document on their computers. Beyond that, participants reported that the Workbook was simple to navigate and use.

“*I think the booklet flowed nicely from one to another, there was a logical progression. So, it made it easier for one conversation to dip into the next topic, the subject.” (Caregiver7).*

#### 3.3.2 Usefulness

Regarding usefulness, many of the participants' already high levels of readiness for and confidence in ACP limited how much additional benefit they could derive from the Workbook. In general, participants' comments related to usefulness reflected what would be expected from effective ACP: improved communication between patient and caregiver, improved communication with clinicians, improved preparation, the opportunity to reflect and clarify.

*“In the emergency room and the ICU last year, they were… pressuring me because [my mother's] situation was really critical. If they didn't intubate her that might be the end of it. I felt so caught, like I didn't know the right answer even though we've had [many] conversations. Going through the Workbook actually was really helpful to me now for getting a sense of what your thinking is now, and you were very clear. That gave me some peace of mind about what you really want now, given the course of your disease.” (Caregiver8 in conversation with her mother, Patient8).*

The main barrier to usefulness was related to participants' uncertainty about how and whether to bring the Workbook to their clinicians. Patients expressed concern that by sharing their workbook they could be seen as “overstepping” or that their clinicians might not have the time and interest to appreciate the information contained within. The following quote relates to both usefulness and safety:

*“If I shared my completed Workbook with [my doctor], would they be critical? Would they think this is too much talking about yourself? It feels like it's being exposed too much to people...It's a two-way thing.” (Patient19).*

#### 3.3.3 Acceptability & Safety

A major theme for both acceptability and safety was the challenge of confronting potentially difficult emotions. Participants acknowledged that this was difficult:

*“You're having to confront unpleasant thoughts. You're having to confront your or your loved one's mortality, and having to think about their or your decline, life without them, that this is shifting. So yes, if you don't have to think of things that are uncomfortable or unpleasant, you much prefer not.” (Caregiver11).*

However, none of the participants regarded this difficulty as a “problem.” Instead, they saw it as part of the process and part of what made the Workbook meaningful:

*“As far as the emotions go— I mean, if it wasn't causing you to feel strong emotions, it probably isn't working right, the way I see it. It needs to be out there, and I felt a lot of relief actually, because I've got all of this stuff down in writing.” (Patient2).*

#### 3.3.4 Who and how the workbook is introduced matters

Participants shared that while they did not think there was an issue with the Workbook itself, this did not guarantee patients would want to use it. This theme arose in response to questions about how to ensure the Workbook was as acceptable as possible to URM.

*“It's not the Workbook itself. It's kind of how do you get people to crack it open in the first place?” (Caregiver6).*

Instead, what was described as more important was who introduced the Workbook and how it was presented. If presented by a trusted person and in a trustworthy manner, it could be acceptable. The implication was that if presented by a person who was not yet trusted or by a trusted person in an untrustworthy manner (e.g. too pushy), patients would be unlikely to engage with it because of the sensitivity and potential difficulty of the subject.

*“…you can't just drop this [Workbook] on them and say, ‘Here you go, fill this out for me’ and expect them to actually do it [and] give you truthful answers. Because you don't talk about this stuff all the time. This is something that people are going to have to really sit down and think about…If you offered to sit and fill it out together, or at least partially— maybe pick out a few questions— the patient would see, well, this physician is making an effort. I know they're busy but they're sitting here asking me about how I feel, and they're taking time out of their day to address me. I think that could be a way that you could gain trust.” (Expert11).*

#### 3.3.5 Readiness to engage in ACP

As noted in the Methods section, we pivoted our recruitment and sampling approach with the goal of improving participant diversity. This allowed recruitment of a more diverse group of participants with regards to race, ethnicity, geography, and age. However, the high level of ACP experience and positive feeling for the process reported by participants reflected that we still primarily reached those who were already “ready” to do or had already done ACP. This finding dovetails with our quantitative finding that the mean Advance Care Planning Engagement Score for our participants was quite high (*M* = 4.28, *SD* = 0.85, range 1–5).

*“Yeah, health care proxy, making the decision around having a DNR, not wanting to be hospitalized again. So, given the fact that we had had that series of what might be called difficult conversations, which really weren't that difficult for us, filling out the workbook was easy.”(Patient7).*

There were some exceptions. Nine participants (31%) who were less “ready” to do ACP consented to participate anyway (mean ACP Engagement Score ranged from 2.21 to 3.78). All nine of those participants described enrolling because they had an alternate reason for participating – either a family member who wanted to participate in the study and needed a dyad partner, or a professional interest in ACP. In general, these participants reported finding the Workbook useful and acceptable despite their lower degree of readiness.

*“I think it's very important to bring it up because someone like me – I'll avoid it forever if it's not brought up, if I'm not made to deal with it. (Caregiver3).*

#### 3.3.6 Recommended changes to workbook

Participants identified multiple areas that could be improved, such as simplifying instructions, addressing caregivers directly, diversifying the imagery on the cover, word changes to broaden relatability, and improving color choices to meet the needs of visually impaired people. Considered changes to the Workbook based on participant feedback and observations are summarized in Supplemental Table. While every recommendation was considered, not all were implemented as most entailed trade-offs. For example, several participants wanted text boxes to explain their responses to the Likert scale questions on page 4. We decided not to add these because it would have increased the length of the Workbook from 8 to 9 pages, because the Likert questions were included for people who might struggle with the open-ended questions (nearly every other question in the Workbook is open-ended), and because we noticed participants writing their clarifying comments in the margins.

## 4 Discussion, innovation, and conclusion

Participants reported that, on the whole, the *What Matters to Me* Workbook is an acceptable, easy-to-use, useful, and safe resource for seriously ill patients and their caregivers. Their experiences and observations allowed us to make multiple improvements in the Workbook, and generated some insights that can be valuable for those using the Workbook and the ACP field.

While the participants tended to have high levels of readiness to engage in advance care planning, this circumstance arose because we asked participants to actually do advance care planning (as opposed to just asking their opinions about the workbook). Thus, we faced the same challenge that clinicians face when trying to encourage advance care planning—patients and caregivers who were not ready did not want to participate, particularly those from underrepresented and minority communities.

Changing the recruitment approach allowed us to recruit a more racially, ethnically, and geographically diverse sample, but as volunteers, these participants continued to be those who were positively predisposed to ACP. In future ACP research, it is important to ensure enrollment of participants who have the motivation to participate other than interest in ACP. In this study, that was accomplished by more enthusiastic patients or caregivers asking their less-enthusiastic family members or friends to participate. An alternative strategy could be to pay volunteers enough that the payment is sufficient motivation to participate. This approach was used effectively by the Massachusetts Coalition for Serious Illness Care to access URM respondents [18] and those who may not be enthusiastic about ACP [19].

The finding that the acceptability of the Workbook is less about the Workbook itself, and more about who is recommending it and how they introduce it echoes the concept of “trusted messengers” [20]. It is easy to imagine how the Workbook might seem acceptable if it is introduced by a trusted clinician or family member as a way to help give them a say in their care. On the other hand, it is also easy to see how, if introduced by a person who is a stranger or not trusted and without sufficient explanation, then the Workbook could be perceived very differently, for example as an indirect way of telling them that they need to prepare for death [21].

Health professionals who enjoy trusting relationships with their patients and their caregivers still need to make sure they personally introduce the Workbook and thoroughly explain what it is and why they are giving it to the patient [22] When a trusting relationship does not exist, as is common in underserved and marginalized communities [[23], [24], [25], [26]], it may be helpful to partner with community organizations such as faith communities who can introduce the Workbook to their congregants [[27], [28], [29]]. This approach needs to be paired with a parallel effort to familiarize clinicians and health systems with the Workbook, because even among our very prepared and confident participant sample, there were worries that their clinicians would not be receptive to their own efforts at ACP. Participants' responses to the ACP-e survey indicated the least readiness to talk to their doctors, however their lack of readiness could also reflect a concern that their doctors were not ready to talk to them.

### 4.1 Innovation

Advance Care Planning in the United States has evolved significantly in the past 20 years, with a number of innovative approaches that have improved on the original “Living Will” concept. However, each innovation exists mostly independently from others. The PREPARE website offers low literacy state specific advance directives, POLST offers portable orders for life sustaining treatment, 5 Wishes offers an expanded advance directive that addresses important issues besides tube feeding and life-support, etc. Some, like the Conversation Project's Conversation Starter Guide, are patient-facing, while others, like Ariadne Lab's Serious Illness Conversation Guide are clinician-facing. The Workbook is a patient-facing tool designed to prepare patients and their important people for a serious illness conversation using the Guide, and as a key element of the Serious Illness Care Program. The Program includes important health system changes like a process for identifying high-risk patients, reminders for conversations, and a template in the electronic health record to document and retrieve conversations.

Thus, the main innovation here is an integrated approach to help prepare patients, their family caregivers, clinicians and health systems to understand and honor what matters most to patients. This becomes especially important when patients lose the ability to make their own decisions as they near the end-of-life. At that point patients, family caregivers, and clinicians need to be prepared for difficult shared decisions as any of the parties can undermine the others' best efforts in the heat of the moment. Because patients are likely to be cared for by many health professionals who are meeting them for the first time, e.g.in the emergency room, hospital, or intensive care unit, the health system must also be prepared by ensuring clinicians working in those areas habitually identify, review, and honor advance care planning and serious illness conversation documentation.

A future direction for the workbook is to create an electronic version. One thing that makes completing documents like the Workbook is that it requires a fair amount of abstract and creative thinking. An electronic version could, by accumulating different people's responses over time, help by using algorithms like those used in retail websites: “people who liked what you like said x, y, and z.” Although that approach can also limit thinking, reviewing a list of different suggestions tailored to other responses could also really help people by introducing ideas they hadn't thought of, or by normalizing ideas that they had but were afraid to express.

### 4.2 Limitations

The inherent bias created by depending on research participants who volunteer for a study is often, but not always [30], noted as a limitation in ACP research and systematic reviews of ACP research [[31], [32], [33]]. In our study, many had already done ACP and completed formal documents, and this unusual degree of preparation created a limit to the usefulness of the Workbook for them.

In addition to the bias created by relying on volunteers and the bias created by including caregiving experts to ensure sufficient racial and ethnic diversity, this study has several other limitations. We were unsuccessful in recruiting participants who identified as part of the LGBTQ+ community. The eight unvalidated investigator-authored items assessing the four dimensions: acceptability, ease of use, usefulness, and safety should be considered experimental.. We chose the four dimensions after reviewing the literature and finding existing evaluation methods missed important considerations. For example, the Patient Education Materials Assessment Tool [34] focuses on understandability and actionability, clearly important but too narrowly focused for our purposes. We also sought to determine acceptability to a diverse group of participants and ensure that we could identify and minimize unintended harms. The limitations of this study suggest that further investigation is necessary to determine the acceptability, ease-of-use-, usefulness, and safety among underrepresented communities, where medical mistrust is high.

### 4.3 Conclusion

If presented in the right way by someone they trust, the *What Matters to Me* Workbook can be an acceptable, easy-to-use, useful, and safe resource for patients with serious illness and their caregivers. Preparing patients and caregivers to answer questions about “what matters most” beforehand could make serious illness conversations with clinicians more effective and efficient, but this needs to be tested in future research.

## Author contributions

All authors approved the final manuscript.

Erik K. Fromme, MD, MCR, FAAPHM: conceptualization, funding acquisition, data acquisition and data analysis, original draft writing, review and editing.

Lauren Nisotel, MPH: conceptualization, project administration, review and editing.

Kimberly Mendoza, MD, PhD, MPH: subject recruitment, data acquisition, review and editing.

Ayush Thacker: subject recruitment, data acquisition, review and editing.

Kurt Lowery, MPH: project administration, review and editing.

Bukiwe Sihlongonyane, MPH: quantitative data management.

Katherine O. DeBartolo: conceptualization, review, and editing.

Jane Roessner, PhD: conceptualization, review and editing.

Judy N. Margo, DrPH, MPH: qualitative data curation and analysis, original draft writing, review and editing.

## Declaration of Competing Interest

The authors declare that they have no known competing financial interests or personal relationships that could have appeared to influence the work reported in this paper.

## Appendix A Supplementary data

Workbook Revisions Based on Participant Feedback. Supplementary material
