# Supplementary material for: Testing the What Matters to Me workbook in a diverse sample of seriously ill patients and caregivers
Source: PEC Innov. 2023 Sep 16;3:100216. doi: 10.1016/j.pecinn.2023.100216 (PMC10523264; doi:10.1016/j.pecinn.2023.100216)
Supplement: Supplementary file 1 — Workbook Revisions Based on Participant Feedback. [file mmc1.docx]

**Supplemental Table 4** *Workbook Revisions Based on Participant Feedback*

| Page # | Original | Exemplar Quote | Revised | Note(s) |
| --- | --- | --- | --- | --- |
| 1 | Cover art consisted solely of individuals. | “I wonder if there was a way to include… LBGQ+?” (Expert10) | Cover art includes 3 pairs, including 2 same-sex couples. | This change also acknowledges caregivers. |
| 1 | Title: *Workbook – What Matters to Me: A Guide to Serious Illness Conversations* | n/a | Title: *What Matters to Me: A Workbook for People with Serious Illness* | None of the participants used the term “serious illness conversation,” so we replaced this technical term with plain language. |
| 2 | Note in callout box gives tips for users completing the document electronically on a computer. | “That box on the right that says “you need to save this document”... most of the time we”re dealing with folks who are older… and that might be a challenge for them.” (Caregiver13) | Note now suggests two ways to complete document: electronically on a computer OR by printing a paper copy and completing it by hand. | Problems with opening and completing the Adobe PDF document electronically were by far the most common and frustrating ease-of-use complaint. |
| 2 | My Health and other headings used Ariadne Labs “goldenrod” color and “Examples” text colored grey. When printed in black & white/greyscale those colors were light grey. | “For a person with visual impairment...this is hard to read.” (Patient19) | Goldenrod changed to dark orange, text in grey font enlarged, **bold emphasis** added to important text; PDF altered so that greyscale prints all text in dark grey or black. | Participant had a neurological illness that affected their vision and couldn’t even see the goldenrod text |
| 2 | Initial instructions were presented as text without emphasis. | n/a | Initial instructions are presented as bullet-pointed actions with bold emphasis such as “**Do the workbook by yourself or with someone else.**” | Several participants suggested adding instructions that were actually already present in the instructions; we concluded that the instructions were insufficiently clear or being glossed over. |
| 2 | Initial version of the Workbook was addressed solely to the patient. | “I think maybe just some language around being thoughtful about how you set up the conversation… I definitely feel like I kind of just dove into it, you know, and like, maybe just like weigh some of the pros and cons of – of who is present and that kind of thing.” (Caregiver14) | Revised version adds a callout box with guidance for caregivers about how to set up the conversation for success, particularly when working with patients who are prone to confusion. | In multiple patient/caregiver pairs, the patient was able to do the Workbook because of their caregiver. |
| 3 | Instructions for Likert scale questions noted there would be a range of feelings and no right or wrong answers. | “… I wanted a little bit more grounding…that there is the full range in between (the two anchors). There are no right or wrong answers.” (Expert15) | Added additional clarification that the scales may change over time and could be useful as conversation starters. | Participants sometimes wrote explanatory comments in the margins of the Likert scale section. |
| 4 | My fears and worries: some of the examples “I don’t want to become dependent” and “I don’t want to be a burden on my loved ones.” | “As the patient (I) have to have some comfort with…knowing who my caregiver is now… if that caregiver should become incapacitated, what are my thoughts about what I’m going to do next?” (Patient2) | Both examples were removed and replaced with a question, “What if I need more care than my caregivers can provide?” | Another caregiver, whose son had severe developmental disabilities, noted that the example, “I don’t want to become dependent,” was uncomfortable to read as he was already dependent. |
| 6 | Final question was “What else do you want to make sure your family, friends, and clinicians  know about you and your wishes and preferences for care if you get sicker?” | n/a - This section was almost always left blank. | The question prompt was changed to: “Is there anything else you want to make sure your family, friends, and  health care team know about you and your wishes and preferences for care if you get sicker?” | We thought the re-phrased question is less burdensome – if there was something not covered, they could add it. If not, they could leave it blank without having to wrack their brains to come up with something else. |
| 7 | My People section began by asking who the patient would want to make medical decisions if they’re not able to. | “…that's really what you're asking …who would you want to (be involved in making) medical decisions? So I would just reflect that up top.” (Expert14) | Instead, section opens with a more inclusive question: “Are there key people who will be involved in your care (family members,  friends, faith leaders, others)?” | This order was much more logical – starting with who the key people are and then moving to who would be trusted to make decisions rather than vice-versa. |
| 8 | Next Steps consisted of 3 bulleted short paragraphs that discussed generally how to initiate a conversation with their health care team. | “… if there was a way to lead the patient to the next steps, which is to say, “Okay, now this has to be kind of formalized, you thought about it, this is great.”…now, the reader should get this written down, so others can have it at the ready.” (Caregiver6) | Next Steps expanded the options and added bold-face specific action items like “**Talk it over with your health care team**” or “**Pick a proxy**.” | As with the instructions at the beginning, people tended to miss the “next steps” at the end, so we tried to make them more noticeable and action oriented. |

*We confirm all patient/personal identifiers have been removed or disguised so the patient/person(s) described are not identifiable and cannot be identified through the details of the story.*
